# Cognitive Decline Over a 5-Year Period: The National Health and Aging Trends Study

**DOI:** 10.1093/geroni/igab046.744

**Published:** 2021-12-17

**Authors:** Allison Gibson, Sarah Holmes, Virgina Richardson

**Affiliations:** 1 University of Kentucky, University of Kentucky, Kentucky, United States; 2 University of Maryland School of Nursing, Baltimore, Maryland, United States; 3 The Ohio State University College of Social Work, Columbus, Ohio, United States

## Abstract

The classification of Alzheimer’s disease and related dementia (ADRD) is important for understanding the progression of cognitive decline. This longitudinal study used data from the National Health and Aging Trends Study (NHATS). A sample of 3,287 eligible Medicare beneficiaries were included in the study. Nine cognitive profiles were examined from Waves 1 to 5 (2011-2015). Discriminant factor analysis was used to identify factors that differentiated across the cognitive profiles. Results showed that 1,076 had some measure of “possible” or “probable” dementia over the 5 years. In Wave 1, there were 104 self-reported ADRD diagnoses, and in Wave 5, there were 327 self-reported ADRD diagnoses. Social participation was an important factor in those that impairment reversed from probable to possible ADRD. Findings support previous evidence that certain activities may slow or reverse cognitive decline and can inform future studies exploring the causality of dementia onset.

